# Feasibility of Wet and Non-Wet Placement of Seprafilm in Laparoscopic Surgeries: A Randomized Controlled Trial

**DOI:** 10.3390/jcm10163526

**Published:** 2021-08-11

**Authors:** Chun-Shuo Hsu, Dah-Ching Ding

**Affiliations:** 1 Department of Obstetrics and Gynecology, Dalin Tzu Chi Hospital, Buddhist Tzu Chi Medical Foundation, Tzu Chi University, Chiayi 622, Taiwan; d850092@gmail.com; 2 Department of Obstetrics and Gynecology, Hualien Tzu Chi Hospital, Buddhist Tzu Chi Medical Foundation, Tzu Chi University, Hualien 970, Taiwan; 3 Institute of Medical Sciences, School of Medicine, Tzu Chi University, Hualien 970, Taiwan

**Keywords:** Seprafilm, apply, wet, randomized, laparoscopy

## Abstract

Seprafilm becomes brittle and sticky after contact with water, rendering it difficult to use in laparoscopic surgery. Hence, Seprafilm is not used frequently in laparoscopic surgery. This prospective randomized controlled trial aimed to compare the feasibility of two methods of application of Seprafilm: wet and non-wet. Two groups comprised 30 patients, each with 180 pieces of Seprafilm. Symptomatic patients who underwent laparoscopic surgeries, including hysterectomy and adnexal surgeries, were recruited. Successful application of Seprafilm was defined as a smooth attachment to the site of application. Sticky and fractured Seprafilm sheets were defined as failed applications. Between March 2016 and December 2017, 60 patients underwent laparoscopic Seprafilm placement. The preparation time was 32.67 ± 16.63 and 79.50 ± 22.01 s in the non-wet and wet groups, respectively (*p <* 0.00). The success rate of application was 95.4% in the non-wet group and 98.3% in the wet group (*p =* 0.09). Placement time was 599.50 ± 90.18 s and 592.53 ± 105.82 s in the non-wet and wet groups, respectively (*p =* 0.25). In conclusion, the wet and non-wet application methods of Seprafilm were feasible in laparoscopic surgeries. The preparation time was different between the two groups. However, the rate of successful application and placement time was not different between the two groups.

## 1. Introduction

Seprafilm (Baxter, Deerfield, IL, USA) is applied for the prevention of post-surgical intra-abdominal adhesions [1,2,3,4]. It is composed of sodium hyaluronate and carboxymethylcellulose and is used as an adhesion barrier. Seprafilm is often used in open abdominal surgery and less frequently in laparoscopic surgery [5]. The characteristics of the Seprafilm material include hard consistency and brittleness during insertion through the trocar, and a sticky consistency after contact with the wet intra-abdominal environment [5]. Therefore, recently, several studies have explored the application of Seprafilm in laparoscopic surgery.

Khaitan et al. reported a technique of inserting Seprafilm during laparoscopic surgery in 2002 [6]. They cut Seprafilm in half and rolled in the sheath, inserted the trocar outside the abdomen, and then put the trocar into the abdomen to apply Seprafilm. In 2006 and 2008, Shinohara et al. reported another novel device to apply Seprafilm in laparoscopic surgery [7,8]. However, this device was not available in other countries. Similarly, Chuang et al. reported a novel technique to apply Seprafilm in 2008 [9]. The technique was different from previous studies because the rolled Seprafilm was inserted directly through the trocar without removing the trocar from the abdominal cavity (non-wet technique). A novel moisturizing Seprafilm technique for laparoscopic surgery (wet technique) was reported by Kusuki et al. [10]. Our group also reported a novel combination technique (combining the Chuang and Kusuki techniques, wet technique) to apply Seprafilm in laparoscopic surgery [11]. Several techniques or devices can be combined in laparoscopic surgery.

To date, there has been no study comparing different techniques for the application of Seprafilm. This randomized controlled study aimed to evaluate the wet technique and non-wet technique as regards placement time and success rate.

## 2. Materials and Methods

### 2.1. Ethics

This randomized clinical study was carried out according to the International Conference on Harmonization Guidelines for Good Clinical Practices and the International Organization for Standardization 14155. The study was approved by the Research Ethics Committee of Dalin Tzu Chi Hospital, Chiayi, Taiwan (IRB B10403023-1).

### 2.2. Patients

**Figure 1:**
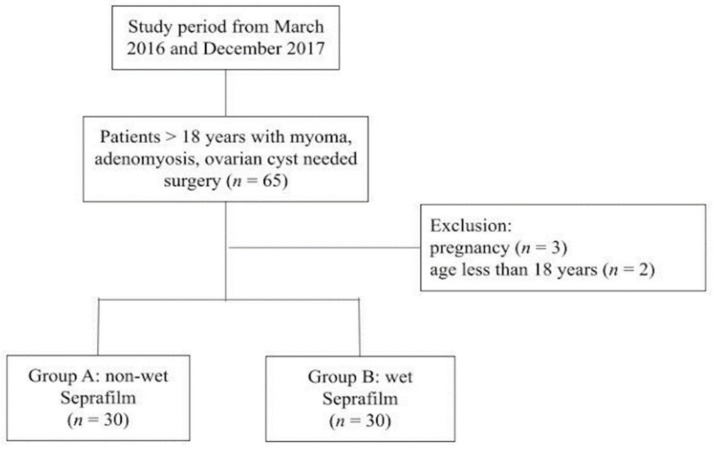
Study flow chart.

The inclusion criterion included women aged >18 with myoma, adenomyosis, or an ovarian cyst, scheduled for laparoscopic surgeries. Patients who wished to undergo placement of Seprafilm to prevent postoperative adhesions were included in the study. During the screening visit (before enrollment), the patients signed an institutional review board-approved informed consent form. Pregnant patients and those aged < 18 years were excluded from the study. The study flow chart is shown in Figure 1. Complete history and physical examination were performed for each patient. This study was conducted in Dalin Tzu Chi Hospital between March 2016 and December 2017

### 2.3. Trial Design and Interventions

This randomized, non-blind, phase 3 trial was conducted at Dalin Tzu Chi Hospital. Envelope-generated randomization was performed at the time of the procedure. Patients were assigned randomly, in a 1:1 ratio, to undergo non-wet and wet Seprafilm placement.

### 2.4. Surgical Technique

All surgical procedures were performed by the same surgeon in the hospital. Seprafilm was cut into six small pieces for placement. Group A was designated as the non-wet group, with a plastic sheet covered with Seprafilm and rolled like a cigar. The roll was then inserted through an 11-mm trocar to the pelvic cavity for placement. Group B is designated as the wet group, with Seprafilm placed onto a wet gauze. Seprafilm was then covered with a plastic sheet and rolled like a cigar, and inserted in the same manner as in Group A. A detailed placement method has been described in previous studies [9,10,11].

### 2.5. Outcomes

The primary outcome was total placement time (divided into preparation and placement time). The secondary outcomes were the successful placement rate and correct placement rate. The definitions of successfully placed Seprafilm were as follows: in total and subtotal hysterectomy, the film framed the endocervical region and bilateral peritoneum opening; in ovarian cystectomy, the film was placed on the lower and upper surface of the ovary; and in myomectomy, the film framed the suture line, 1 cm away from the line. If Seprafilm was successfully placed onto the surgical surface but did not fit the above criteria, it was characterized as incorrect placement.

### 2.6. Statistical Methods

We calculated that having 30 patients in each group (estimated failure rate in the wet group (5%) than in the non-wet group (33%)) would provide the trial with 80% power, at a two-sided significance level of 0.05. Data are presented as the mean ± standard deviation (SD) or number/percent (*n* (%)) [11]. Random allocation was performed using a random balanced table, and treatment allocation was determined by the operating room nurse via blinded, numbered, and sealed envelopes. Quantitative variables were compared using a *t*-test (parametric) or non-parametric Mann–Whitney–Wilcoxon test. Pearson coefficients were computed to evaluate the correlation between quantitative variables. Categorical data were described by the absolute and relative (%) frequencies and 95% CI. Proportions were compared using a chi-square test or a Fisher exact test if the assumptions of the chi-square (theoretical frequency < 5) were not met. Statistical analysis was performed using SAS software version 8.2 and Minitab version 15.0, using an Error Type I of 5%.

## 3. Results

Sixty patients receiving Seprafilm placement from March 2016 and December 2017 were recruited for this study (Figure 1). Group A included 30 patients who underwent non-wet Seprafilm placement, and group B included 30 patients who received wet Seprafilm placement. There was no significant difference in terms of patient age, body mass index, parity, and surgical time between the groups at baseline (Table 1). The hospital stay was 4.2 ± 1.1 and 4.9 ± 1.2 in group A and group B, respectively (*p =* 0.04). The distribution of surgery types was different between the two groups (*p =* 0.027). LAVH consisted of 60% of group A, and adnexal surgery consisted of 53.3% of group B.

The characteristics of Seprafilm placement are listed in Table 2. The difference in preparation time was significant between the two groups (32.67 ± 16.63 s and 79.50 ± 22.01 s in groups A and B, respectively, *p <* 0.00). However, the other parameters, such as placement time, successful placement rate, correct placement rate, and failure rate, were not significant (Table 2).

The difference in operating times between failed and successful cases is listed in Table 3. There was no significant difference in operating time between failed and successful cases (172.3 ± 43.7 s vs. 146.2 ± 47.8 s, *p =* 0.08).

Table 4 lists the characteristics of failed cases in the two groups. There was no significant difference in age, parity, BMI, operating time, and operative types in the failed cases of the two groups. The wet group’s preparation time and hospital stay were significantly longer than those of the non-wet group (*p* = 0.02 and *p =* 0.008 in the preparation time and hospital stay, respectively). There were no postoperative complications related to the adoption of Seprafilm.

## 4. Discussion

We compared wet and non-wet techniques for Seprafilm application in laparoscopic surgery. The preparation time was 32.67 ± 16.63 s and 79.50 ± 22.01 s in the non-wet group and 98.3% in the wet group (*p <* 0.00). The success rate of application was 95.4% in the non-wet group and 98.3% in the wet group (*p =* 0.09). Placement time was 599.50 ± 90.18 s and 592.53 ± 105.82 s in the non-wet and wet groups, respectively (*p =* 0.25). The results show that the outcomes were comparable between the two groups, except for preparation time.

Recently, Ota et al. reported a new reducer application method (non-wet) for applying Seprafilm [12]. Their application time was 5.2 and 4.8 min for two surgeons, respectively. They cut one piece of Seprafilm into four pieces and folded them together into the reducer. They saved the time of preparation. Chuang et al. also reported folding two pieces of Seprafilm (non-wet) together to save time [13]. The mean time of application time was 8 min. Weng et al. also reported a novel method of exposing Seprafilm for 5 min (wet-similarity) before insertion for softening purposes and cutting Seprafilm into four pieces [14]. They inserted Seprafilm pieces one at a time. The average application time was 4 min for four pieces. Our group also reported a novel combination technique (wet) to apply Seprafilm in laparoscopic surgery [11]. The mean placement time was 4.4 and 3.4 min in the single port and multiport surgeries, respectively. In this study, we cut Seprafilm into six pieces and rolled it into a plastic sheet before inserting it into the abdominal cavity. The preparation time of the wet method was significantly longer than that of the non-wet method due to the additional time of exposure to wet gauze. The preparation time was 79.50 ± 22.01 s and 32.67 ± 16.63 s in the wet and non-wet groups, respectively. The application time was 592.53 ± 105.82 s and 599.50 ± 90.18 s in the wet and non-wet groups, respectively. The application time seemed longer (nearly 10 min) than that in previous studies.

Due to the tendency of Seprafilm to break easily when dry and to become sticky when wet, the success rate of application cannot reach 100% easily. In our previous study (wet technique), the success rate of application in laparoscopic surgery could reach 95.7% (92.3% in single port and 100% in multiport) [11]. We suggested that failure in single-port surgery was due to the difficult manipulation of Seprafilm during application. In the Ota et al. study (non-wet), the application success rate was 100% for two doctors [12]. They used a reducer to protect Seprafilm. Weng et al. (wet) reported a success rate of 97.8% [14]. They found that they could not insert a uterine manipulator due to an intact hymen (9 of 11 failure cases), which may have been the cause of failure. Kusuki et al. reported a novel moisturizing Seprafilm technique for laparoscopic surgery (wet) [10]. In their study, a 100% success rate and an 80% success rate in insertion and placement, respectively, were obtained. In this study, the success rate of application was 98.3% and 95.4% in the wet group and non-wet group, respectively. We speculated that the cause of failure was the duration of the operation. A longer operation caused a wetter environment, which may have caused failure in the application of Seprafilm. 

Regarding the length of hospital stay, Weng et al. (wet) reported the same 2 days in the successful and failed placement groups [14]. They only studied the length of hospital stay in laparoscopic myomectomy patients. In this study, hospital stays of failure cases were longer in the wet group than in the non-wet group (Table 4). The type of operation may affect the hospital stay. However, there were no significant differences in the operation type between the two groups of the failure cases. We speculated that the distribution of operation type in the two groups might affect the hospital stay. The number of LAVH was more in group A, and adnexal surgery was more in group B. Nevertheless, the hospital stay paid by insurance was the same (4 days) among the three kinds of operations. The same trends were observed in both patients of the two groups. Therefore, a further large-scale study is required to analyze the cause of this phenomenon.

The strengths of this study include those associated with prospective projects and a randomized design. Moreover, a standardized surgical technique was performed by one surgeon throughout the study. However, the small sample size was a weakness of this study.

## 5. Conclusions

In conclusion, the wet and non-wet application methods of Seprafilm were feasible in laparoscopic surgeries. The outcome (successful placement rate and placement time) of Seprafilm application with the wet method is comparable to that of the non-wet method, except for preparation time. A larger sample of cases and a longer follow-up duration are needed to confirm our findings.

## Figures and Tables

**Table 1:** Basic characteristics.

	A (Non-Wet) (*n* = 30)	B (Wet) (*n* = 30)	*p* Value
Age	44.2 ± 6.8	41.1 ± 5.6	0.063
Body mass index (kg/m^2^)	24.0 ± 4.0	24.2 ± 3.9	0.804
Parity	1.5 ± 1.1	1.2 ± 1.0	0.291
Surgical time (minute)	161.3 ± 58.3	142.3 ± 32.5	0.125
Hospital stay (days)	4.2 ± 1.1	4.9 ± 1.2	0.04
Operation type			0.027
LAVH	18 (60%)	11 (36.7%)	
LM	6 (20%)	3 (10%)	
Adnexa surgery	6 (20%)	16 (53.3%)	

Data are the Mean ± SD, SD: standard deviation; LAVH: laparoscopically assisted vaginal hysterectomy; LM: laparoscopic myomectomy.

**Table 2:** Seprafilm placement characteristics.

	A (Non-Wet) (*n* = 30)	B (Wet) (*n* = 30)	*p* Value
Preparation time (second)	32.6 ± 16.6	79.5 ± 22.0	<0.001
Placement time (second)	599.5 ± 90.1	592.5 ± 105.8	0.7
Successful placement rate	95.% (171/180)	97.70% (176/180)	0.09
Correct placement rate	93.80% (169/180)	96.60% (174/180)	0.2
Failure rate	30% (9/30)	13% (4/30)	0.2

Data are the Mean ± SD or percentage, SD: standard deviation.

**Table 3:** Operative time difference between failure and success cases.

Operative Time	*n*	Mean	SD	F Value	*p* Value
Failure	13	172.3	43.7	3.15	0.08
Success	47	146.2	47.8		

SD: standard deviation.

**Table 4:** Characteristics of failure cases in the two groups.

	Group A (Non-Wet)	Group B (Wet)	*p* Value
N	9	4	
Age	46 ± 6.8	43.3 ± 5.7	0.5
Parity	1.7 ± 1.0	0.7 ± 0.9	0.1
BMI	24.4 ± 5.0	23.6 ± 3.9	0.7
Operative time (minute)	169.4 ± 48.5	178.8 ± 35.6	0.7
Hospital stay (day)	4 ± 0.8	5.7 ± 0.9	0.008
Preparation time (second)	39.4 ± 26.8	86.5 ± 32.9	0.02
Placement time (second)	638.5 ± 88.3	691.5 ± 97.8	0.3
Operation type			0.1
LAVH	5	0	
LM	2	1	
Adnexa surgery	2	3	

BMI: body mass index; LAVH: laparoscopically assisted vaginal hysterectomy; LM: laparoscopic myomectomy.

## Data Availability

The original data can be provided by the corresponding author upon reasonable request.
